# Supplementary figures and images for: weg2vec: Event embedding for temporal networks
Source: Sci Rep. 2020 Apr 28;10:7164. doi: 10.1038/s41598-020-63221-2 (PMC7189270; doi:10.1038/s41598-020-63221-2)

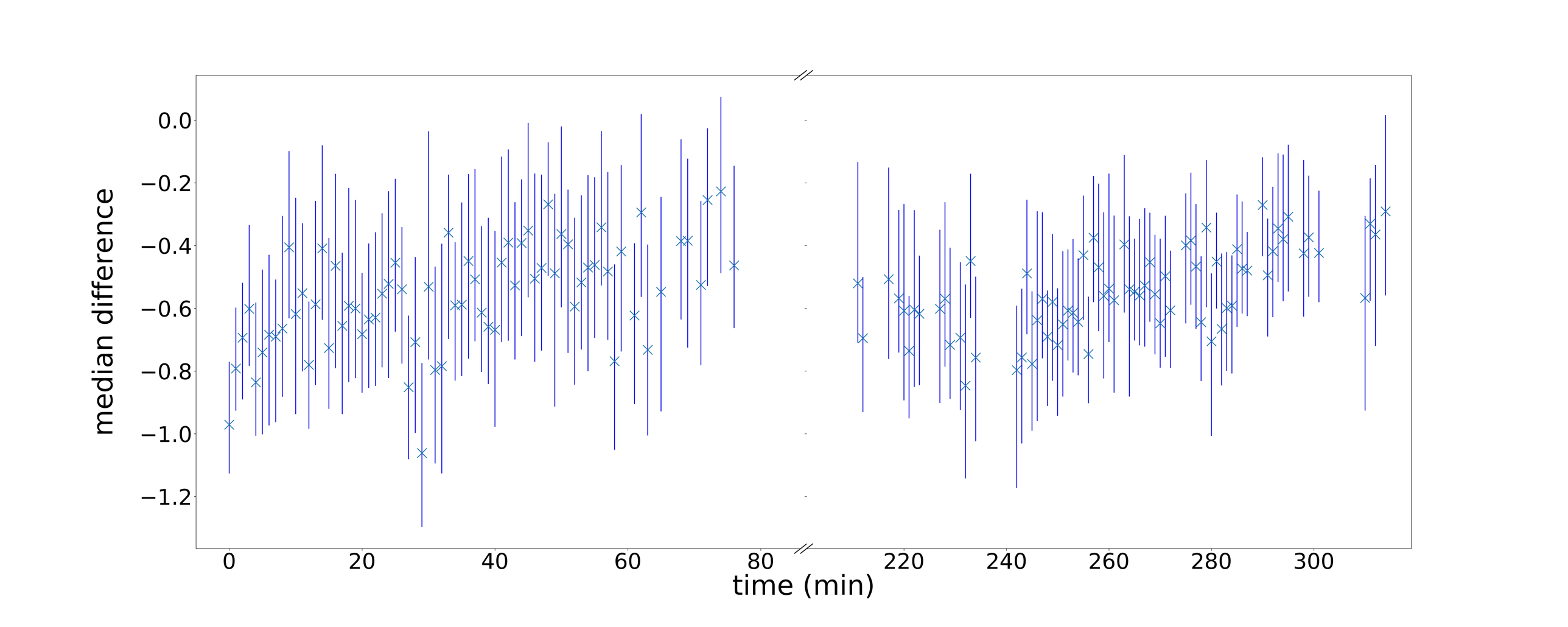

Supplement: Supplementary file 4 — Supplementary Information4. [file 41598_2020_63221_MOESM4_ESM.jpeg]

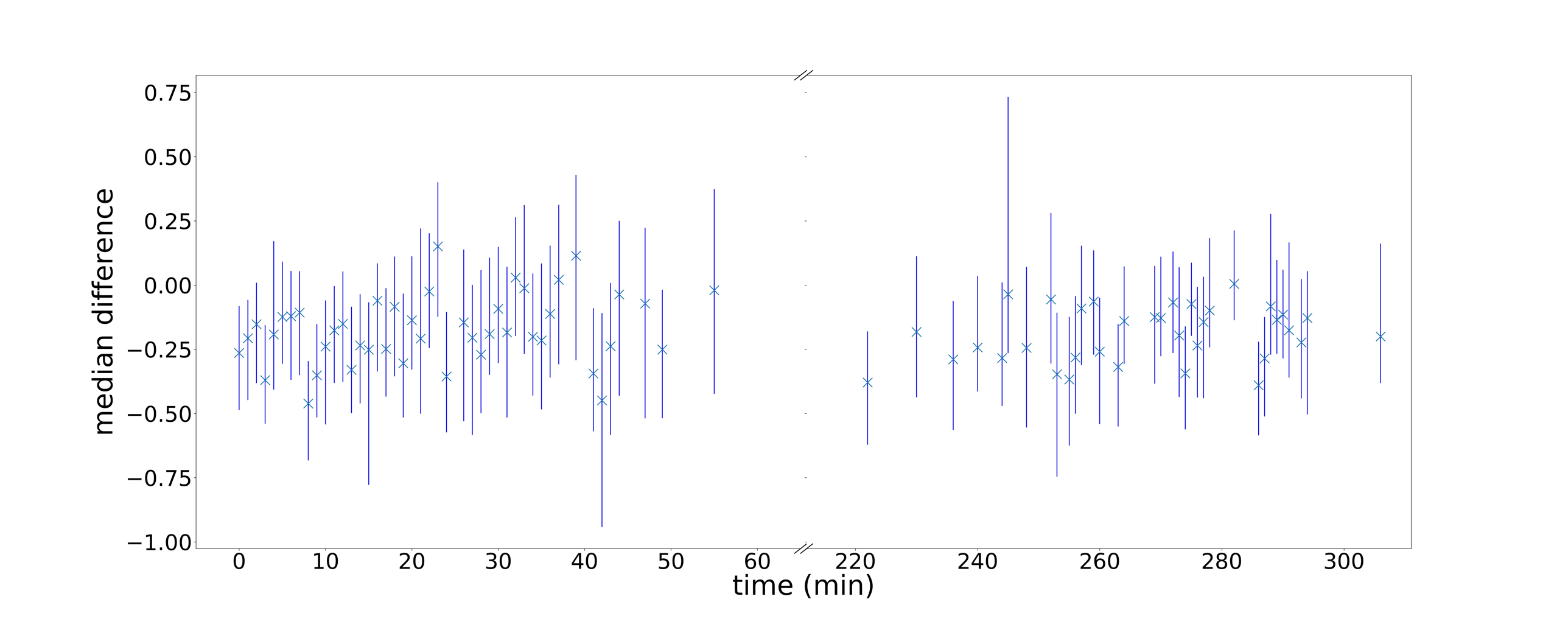

Supplement: Supplementary file 5 — Supplementary Information5. [file 41598_2020_63221_MOESM5_ESM.jpeg]

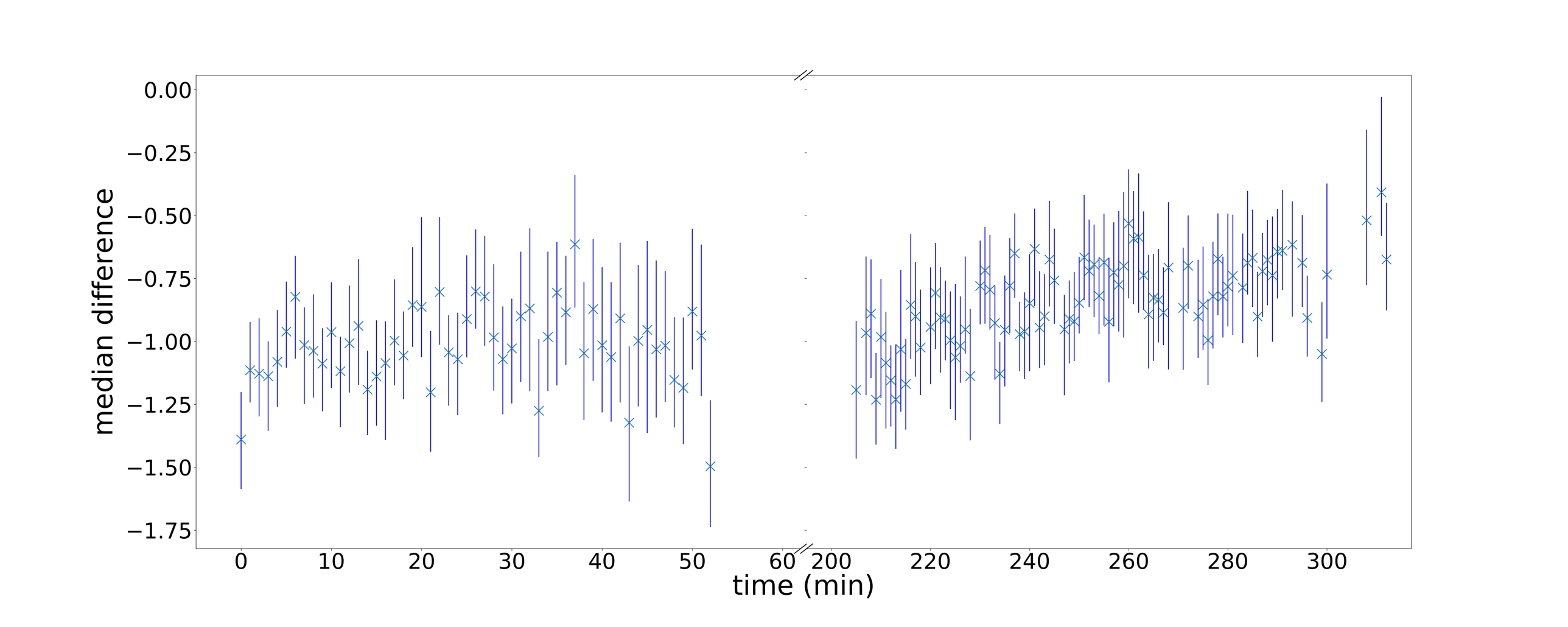

Supplement: Supplementary file 6 — Supplementary Information6. [file 41598_2020_63221_MOESM6_ESM.jpeg]

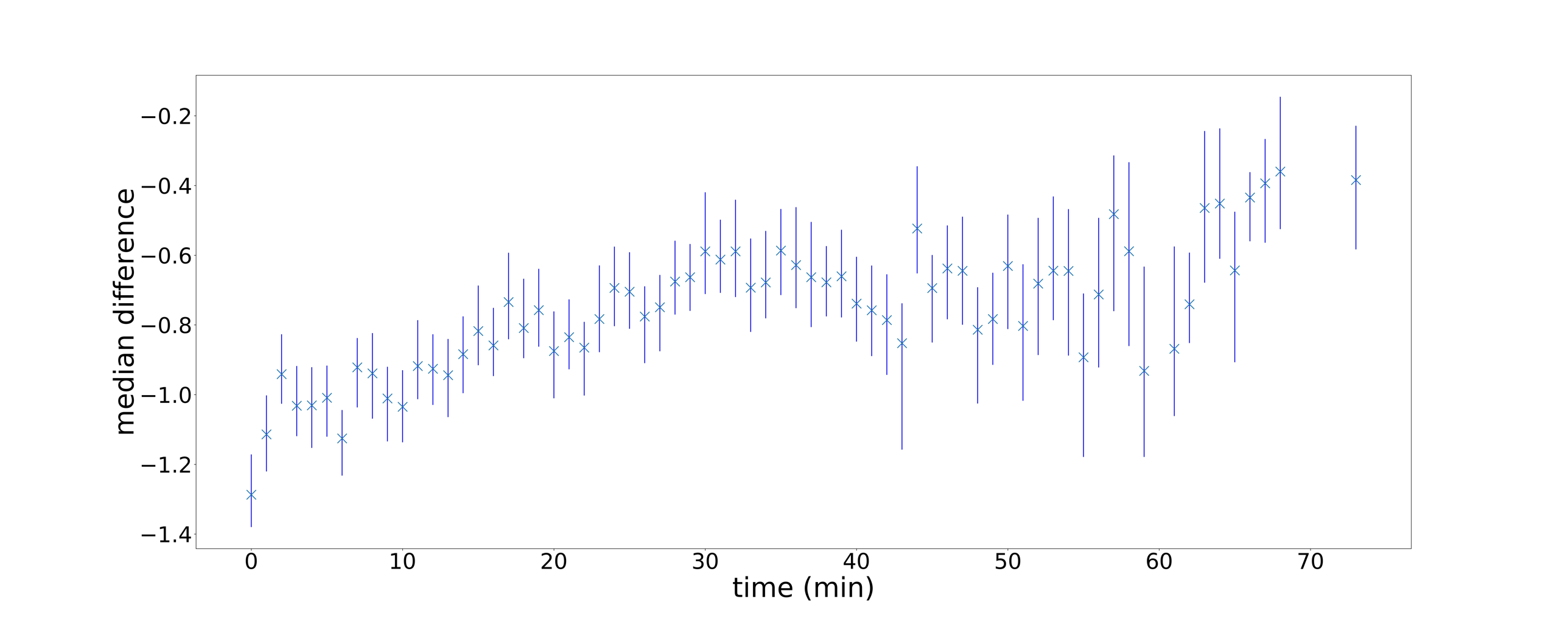

Supplement: Supplementary file 7 — Supplementary Information7. [file 41598_2020_63221_MOESM7_ESM.jpeg]
